# Traditional Chinese Medicine for Essential Hypertension: A Clinical Evidence Map

**DOI:** 10.1155/2020/5471931

**Published:** 2020-12-19

**Authors:** Yan Zhang, Biqing Wang, Chunxiao Ju, Lu Liu, Ying Zhu, Jun Mei, Yue Liu, Fengqin Xu

**Affiliations:** ^1^Graduate School of China Academy of Chinese Medical Sciences, Beijing 100700, China; ^2^Center of Geriatrics Diseases, Xiyuan Hospital, China Academy of Chinese Medical Sciences, Beijing 100091, China; ^3^Cardiovascular Disease Team, China Center for Evidence-Based Medicine of TCM, Beijing 100091, China; ^4^Graduate School of Beijing University of Chinese Medicine, Beijing 100029, China; ^5^Center of Cardiovascular Diseases, Xiyuan Hospital, China Academy of Chinese Medical Sciences, Beijing 100091, China

## Abstract

We systematically retrieved and summarised clinical studies on traditional Chinese medicine (TCM) for the prevention and treatment of essential hypertension (EH) using the evidence map. We aimed to explore the evidence distribution, identify gaps in evidence, and inform on future research priorities. Clinical studies, systematic reviews, guidelines, and pathway studies related to TCM for the prevention and treatment of EH, published between January 2000 and December 2019, were included from databases CNKI, WanFang Data, VIP, PubMed, Embase, and Web of Science. The distribution of evidence was analysed using text descriptions, tables, and graphs. A total of 9,403 articles were included, including 5,920 randomised controlled studies (RCTs), 16 guidelines, expert consensus and path studies, and 139 systematic reviews (SRs). The articles publishing trend increased over time. This study showed that the intervention time of TCM was concentrated at 4–8 weeks, mainly through Chinese herbal medicine (CHM) for the prevention and treatment of elderly hypertension and the complications. A Measurement Tool to Assess Systematic Reviews (AMSTAR) scores of the included reviews ranged from 2 to 10. Most of the SRs had a potentially positive effect (*n* = 120), mainly in 5–8 score. Primary studies and SRs show potential benefits of TCM in lowering blood pressure, lowering the TCM syndrome and symptom differentiation scores (TCM-SSD scores), improving the total effective rate, and reducing the adverse events. The adjunctive effect of TCM on improving the total effective rate, lowering the blood pressure, lowering the TCM-SSD scores, and lowering the adverse effects was only supported by low-quality evidence in this research. The evidence map was used to show the overall research on TCM for the treatment of EH; however, due to the existing problems of the primary studies, the current research conclusion needs further research with higher quality and standardisation.

## 1. Introduction

Hypertension has become a primary global disease and is an important global public health challenge [1]. According to literature, in 2000, 26.4% of adults worldwide suffered from high blood pressure. It is estimated that by 2025, 29.2% of people in the world will suffer from high blood pressure [2]. There is currently an upward trend of the hypertension prevalence and mortality rates among Chinese residents and it is predicted that by 2030, the annual economic burden of cardiovascular disease deaths caused by hypertension in China will reach $6–9 million [3]. A prospective epidemiological study of 47,000 residents in 115 urban and rural communities in China showed that the prevalence rate, awareness rate, treatment rate, and control rate of hypertension in China were 41.9%, 41.6%, 34.4%, and 8.2%, respectively, indicating that prevention, detection, treatment, and control of hypertension should be prioritised [4].

Antihypertensive therapy is currently widely used; however, its understanding, management, and control are not well known due to the adverse effects and intolerance of antihypertensive drugs that the patients currently face [5]. Therefore, more attention must be given to complementary and alternative medical treatments. Systematic reviews (SRs) have shown that traditional Chinese medicine (TCM) has a significant effect on lowering blood pressure but there is little research on its underlying intervention mechanisms [6, 7].

As an evidence integration method, evidence mapping can integrate evidence of various study types under a research topic and comprehensively demonstrate the problems in the research topic, thereby depicting a complete picture of the research field [8–10]. Several evidence mapping reports have been published on the Chinese medical fields such as acupuncture, Tai Chi, massage, and angelica; however, they only included randomised controlled studies (RCTs) and SRs [11–14]. However, the clinical evidence for the prevention and treatment of hypertension by TCM is unclear. Therefore, this study used an evidence map to systematically find relevant literature (observational studies, RCTs, SRs, guidelines, and expert consensus) on the clinical prevention and treatment of essential hypertension, in order to better understand the distribution of evidence in this field, identify gaps in evidence, and provide potential information for priority areas.

## 2. Methods

### 2.1. Database and Search Strategies

The literature searches were conducted using PubMed, Web of Science, Embase, Chinese National Knowledge Infrastructure (CNKI), Chinese Scientific Journal Database (VIP), and WanFang data. The search was restricted from January 1, 2000, to December 31, 2019. We searched the Chinese database using “hypertension”. The retrieval subjects are limited to TCM, integrated Chinese and Western medicines, TCM internal medicine, surgery of Chinese medicine, gynaecology of Chinese medicine, paediatrics of Chinese medicine, and other TCM-related subjects. English database retrieval was divided into two parts. The search terms for the first retrieval included: (“hypertension” OR “blood pressure, high” OR “blood pressures, high” OR “high blood pressure” OR “high blood pressures”) AND (“medicine, Chinese traditional” OR “traditional Chinese medicine” OR “traditional medicine, Chinese” OR “Chinese medicine, traditional” OR “herbal medicine” OR “drugs, Chinese herbal” OR “herbal formula” OR “Chinese herbal medicine” OR “Chinese herb therapy” OR “Chinese herb” OR “herb therapy” OR “herbal remedy” OR “acupuncture”). The second retrieval search term was “hypertension” + hypertension-related formulas and nondrug therapy that frequently appeared in the meta-analysis in the Chinese database; the two retrievals were combined. The literature searched included academic journals, graduation theses, and conference papers.

### 2.2. Inclusion Criteria

The inclusion criteria were as follows:

1. Type of study: RCTs, nonrandomised controlled trials (non-RCTs), cohort studies, case-control studies, cross-sectional studies, real-world studies (RWS), systematic reviews, meta-analyses, expert consensus, guidelines, and clinical pathway studies on TCM intervention for hypertension
2. Type of participants: the patients that met the diagnostic criteria of essential hypertension. There was no limitation on the age, sex, race, time of onset, and cases of the source
3. Type of intervention: TCM (Chinese herbal medicine (CHM) (decoction, tablet, pill, powder, granule, capsule, oral liquid, or injection), nondrug therapy (acupuncture, qigong, massage, and Baduanjin, etc.)), nursing of TCM, or above measures combined with conventional Western medicine that was used in the treatment groups. The comparison interventions were conventional Western medicine, placebo, or blank controls
4. Type of outcome: the main outcomes included blood pressure (BP), total effective rate, TCM syndrome and symptom differentiation (TCM-SSD) scores, and adverse events. TCM prevention and treatment, TCM syndrome type, and duration of TCM intervention

### 2.3. Exclusion Criteria

(1) Clinical experience, (2) clinical trial protocols, (3) meeting abstracts, (4) no full-text, (5) redundant publication, and (6) fundamental researches were excluded.

### 2.4. Literature Screening and Data Extraction

Four authors independently conducted the literature search, study selection, and data extraction, and 2 authors conducted it as a group. The extracted data included the following: (1) basic information: author, publication year, study object and disease, intervention measures, total sample size, and outcome indicators; (2) study type ((i) intervention study: RCTs, non-RCTs, (ii) observational study: a cohort study, case-control study, and cross-sectional study, (iii) secondary study: SRs, guidelines, and clinical pathway studies, (iv) RWS); (3) treatment categories (CPM, CHM, nursing of TCM, acupuncture, massage, TCM exercise therapy, auricular point, acupoint application, multimethod combination, and others); (4) complicating diseases (cerebral haemorrhage, cerebral infarction, angina pectoris/myocardial ischaemia, arrhythmia, diabetes/abnormal glucose metabolism, cardiac insufficiency, anxiety and depression, renal diseases, eye diseases, insomnia/sleep disorders, hyperlipidaemia, hyperuricaemia, metabolic syndrome, atherosclerosis, etc.); and (5) the duration of therapeutic intervention. Disagreements were resolved by discussion, and a consensus was reached through a third party (J. Mei).

### 2.5. Quality Assessment of the Included Systematic Reviews

A Measurement Tool to Assess SRs (AMSTAR), which consists of 11 items, was used to evaluate the methodological quality of all the included SRs. For each item, “Yes,” “No,” “Can't answer,” or “Not applicable” was assigned according to judgement criteria of AMSTAR. The number of “yes” was counted as the total AMSTAR score. A score of 4 or less was considered low quality, a score of 5 to 8 was medium quality, and a score of 9 or more was high quality [15, 16]. Based on the SRs' clinical effectiveness, it was further divided into 4 categories: “evidence of no effect,” “unclear evidence,” “evidence of a potentially positive effect,” and “evidence of a positive effect” [13]. The category “evidence of no effect” meant that the effect of the control group is equal to or better than that of the TCM observation group. “Unclear evidence” meant that the result of a systematic review of similar contents is controversial, or the evidence is summarised as inconclusive by the original study's author. “Evidence of a potentially positive effect” referred to the systematic review of all included clinical studies, combined results, and statistical evidence to show effectiveness but the lack of basic and auxiliary evidence made it difficult to produce positive and reliable conclusions. “Evidence of a positive effect” meant that statistics showed that TCM therapy had a significant effect and that the authors of the systematic review had no major doubts regarding the current evidence and recommend the therapy.

### 2.6. Data Analysis and Presentation

EXCEL 2013 was used to integrate and process the data. The data summary and analysis are shown as text and charts. The distribution of the development trend is depicted as a line chart, the distribution of category proportions as a pie chart, and the distribution of evidence as bubble plots and heatmap.

## 3. Results

### 3.1. Description of the Included Trials

**Figure 1:**
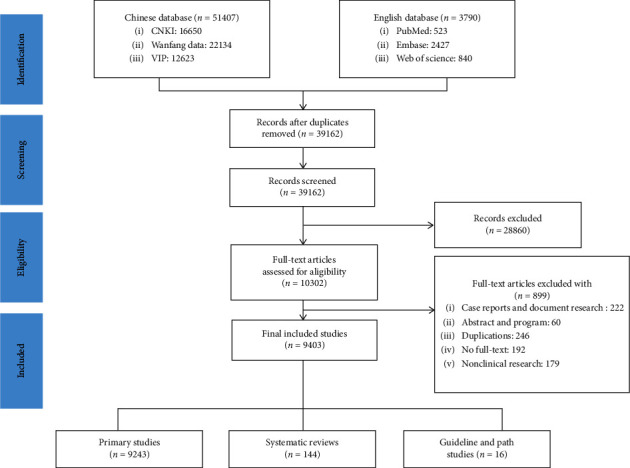
Study flow diagram.

The initial search retrieved 55,197 articles from the six databases. After removing duplicates, 39,162 trials were identified. After screening the titles and abstracts, 10,302 trials were retained. By browsing the full-text articles, we further excluded 899 records. In the end, 9,403 studies were reviewed, including primary studies (*n* = 9,243), systematic reviews (*n* = 144), and guidelines, expert consensus, and path studies (*n* = 16) (Figure 1).

### 3.2. Trends in Publication Year of Clinical Studies

**Figure 2:**
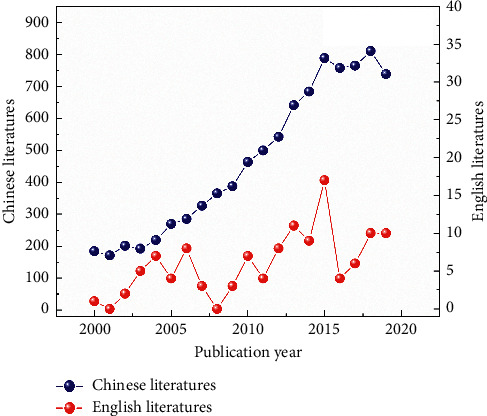
Annual trends in the clinical research literature. The blue line denotes the number of Chinese literature, and the red line denotes the number of English literature.

A total of 9,403 studies were included from January 2000 to December 2019. The number of studies showed an overall rising trend with a peak in 2018 at home and 2015 abroad, respectively (see Figure 2). The TCM role is increasingly being suspected in the prevention and treatment of hypertension, both in China and worldwide.

### 3.3. Type and Scale of Clinical Studies

The clinical studies were mainly RCTs, including intervention studies (RCTs (*n* = 5,920, 63.0%), non-RCTs (*n* = 2,133, 22.7%)), observational studies (*n* = 1185, 12.6%), RWS (*n* = 5, 0.1%), and SRs (*n* = 144, 1.5%). The minimum sample size of the RCT was 10 and the maximum was 2,110 [17]. The maximum sample size of the observational study was 154,083 cases [18], and the sample size of the interventional study was mostly in the range of 60 to 100 cases. In RWS, the sample size ranged from 1,544 to 30,034 cases [19] (see Table 1).

### 3.4. Research on Syndrome and Constitution

**Figure 3:**
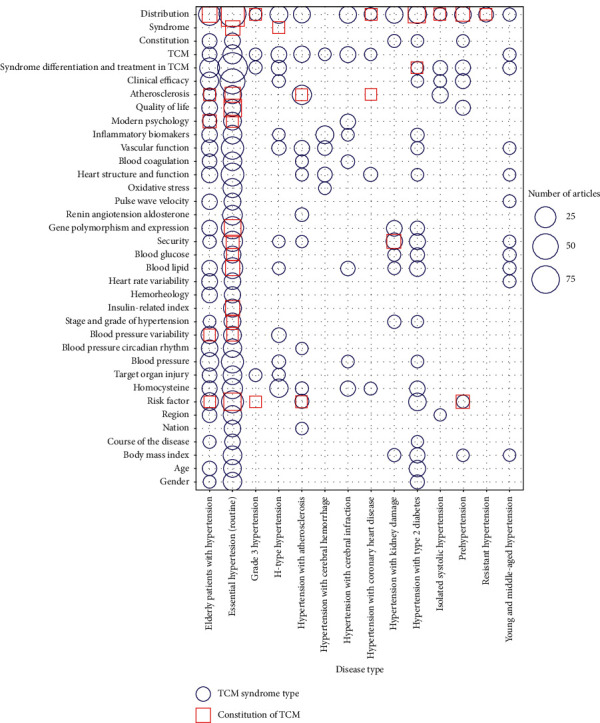
Evidence distributions of clinical studies on syndrome and constitution. Objects of study (*x*-axis) and research content (*y*-axis). The red square indicates the constitution of TCM and the blue bubbles indicate the TCM syndrome types.

A total of 848 clinical studies on TCM syndromes of hypertension were included, of which the syndrome distribution ranked first with a total of 162; others included hypertension syndromes and clinical indicators in young and middle-aged people (*n* = 4) [20–23], syndromes in elderly hypertension (*n* = 124), hypertension stages and grades (*n* = 9) [24], four diagnosis information and TCM syndromes (*n* = 1) [25], and TCM syndromes and clinical indicators in grade 3 hypertension (*n* = 2) [26, 27]. Regarding comorbidity, there were 2 cases of hypertension with arrhythmia, 22 cases of atherosclerosis, 9 cases of a cerebral haemorrhage, 19 cases of cerebral infarction, and 32 cases of diabetes. Studies on the correlation between syndromes and clinical indicators mainly involved indicators such as homocysteine, blood lipid, blood glucose, vascular function, and inflammation. A total of 245 studies on the TCM constitution of hypertension were included, of which there were 12 constitution and syndrome types, mainly involving the phlegm-dampness syndrome [28]. As the syndrome type and constitution articles involved more than 100 kinds of clinical indicators, only the first 36 indicators were shown (see Figure 3).

The bubble plot shows the syndrome and constitution and mainly on a wide range of hypertension and elderly patients with hypertension; however, there are few studies on prehypertension and hypertension grades.

### 3.5. Categories of TCM Prevention and Treatment

**Figure 4:**
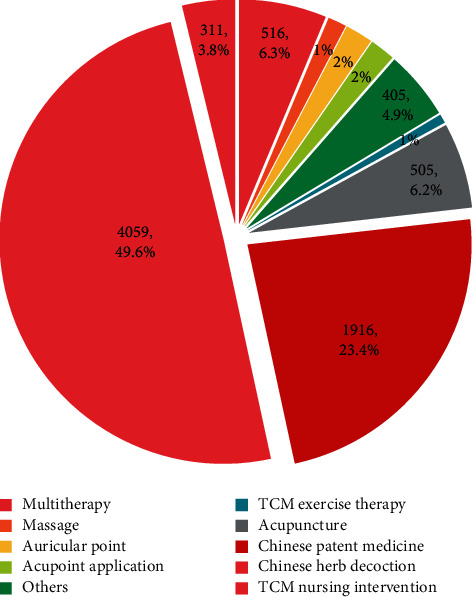
Category distribution of the prevention and treatment of hypertension by TCM.

TCM prevention and treatment schemes are mainly divided into 10 categories, including CHM decoction (*n* = 4,059, 49.6%), Chinese patent medicine (*n* = 1,916, 23.4%), acupuncture ((electroacupuncture and meridional acupuncture) (*n* = 505, 6.2%)), massage (*n* = 109, 1.3%), auricular point and auricular acupuncture (*n* = 163, 2.0%), acupoint application (*n* = 149, 1.8%), TCM exercise therapy (Tai Chi, Baduanjin, wu qinxi) (*n* = 52, 0.6%), TCM comprehensive nursing (*n* = 311, 3.8%), multitherapy (*n* = 516, 6.3%), and others (pediluvium, fumigation, etc.) (*n* = 405, 4.9%)) (Figure 4). CHM and nondrug therapies are widely used for treating hypertension.

### 3.6. Clinical Evaluation of the TCM Treatment Schemes

**Figure 5:**
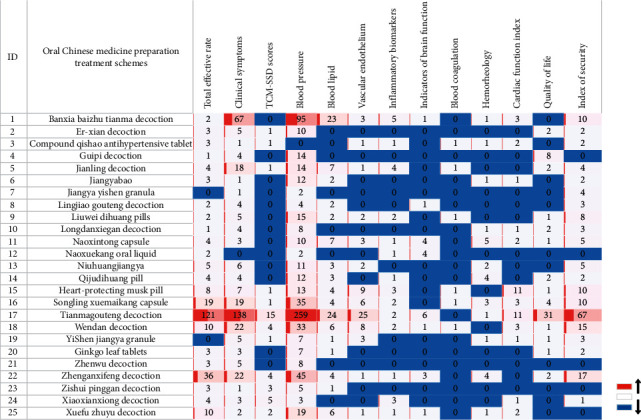
Distribution of clinical evidence for the prevention and treatment of hypertension by oral Chinese herbal preparations. The change of “blue-white-red” colour represents the number of research literature from low to high, and numbers represent the corresponding number of literature. The evaluation index of clinical research is in *x*-axis and oral Chinese medicine preparation is in *y*-axis.

**Figure 6:**
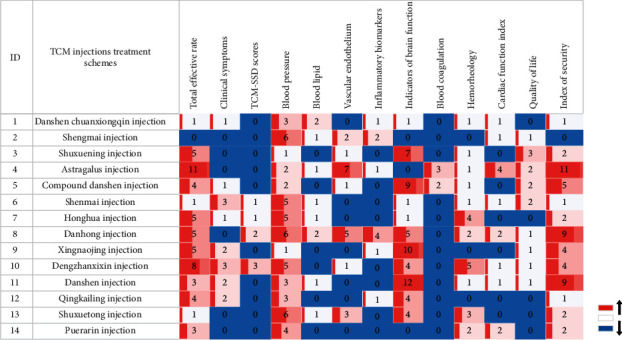
Distribution of clinical evidence on the prevention and treatment of hypertension by TCM injections. The change of “blue-white-red” colour represents the number of research literature from low to high and numbers represent the corresponding number of literature. The evaluation index of clinical research is in *x*-axis and traditional Chinese medicine injection is in *y*-axis.

Regarding hypertension and the complications, more than 5 separate evaluations of clinical studies included 14 injections such as compound Danshen injection, astragalus injection, Shengmai injection, and Danhong injection; 12 oral CPM such as the Niuhuang Jiangya pill, Liuwei Dihuang pill, and Songling Xuemaikang capsule; and 13 types of oral CHM decoctions such as Xuefu Zhuyu decoction, Banxia Bbaizhu Tianma decoction, and Lingjiao Gouteng decoction (Figures 5 and 6). The main treatment methods included the calming liver-yang method, resolving phlegm and quenching wind, promoting blood circulation, and removal of blood stasis. The combination modes were mostly CHM decoction in combination, CHM decoction combined with CPM, and integrated Chinese and Western medicines.

The evaluation of TCM prevention and treatment schemes was divided into several types of indicators: total effective rate, BP, TCM-SSD scores, clinical symptoms, blood lipid levels, inflammatory indicators (e.g., C-reactive protein, inflammatory factors, etc.), brain function evaluation indicators (e.g., National Institute of Health stroke scale (NIHSS), neurological function score, cerebral haematoma absorption, etc.), cardiac function indicators (such as myocardial injury markers, cardiac structure indicators, cardiac function classification, etc.), hemorheology (e.g., blood viscosity, blood flow velocity, etc.), QOL (e.g., SF-36 quality of life scale, etc.), and safety evaluation (e.g., adverse events, rebleeding event, liver and kidney function). The research evidence distribution of commonly used oral CHM preparations and traditional Chinese medicine injections for the prevention and treatment of hypertension is shown in a heatmap (Figures 5 and 6).

Studies of CHM injection and oral traditional Chinese medicine preparations showed that the evaluation indexes of hypertension were mostly related to complications. The total effective rate, BP, brain function evaluation indicators, and safety index of TCM injection had a high degree of attention, shown in red. Blood coagulation and TCM-SSD scores had low attention, shown in blue. The total effective rate, clinical symptoms, BP, and safety index of oral Chinese medicine preparation were highly relevant in the clinical studies. The indicators of blood coagulation, hemorheology, and brain function received little attention, and the research directions were generally consistent.

### 3.7. Investigation of the Application of TCM Prevention and Treatment Schemes

RWS found that the 30,034 hypertension patients in 16 AAA-grade hospitals were mainly treated with intravenous drugs, among which the 3 traditional Chinese medicine preparations, the Danhong injection, Shuxue Ning injection, and Ginkgo Biloba extract, were used more than 10% of the total drugs used [19]. The Beijing Hospital found that Liuwei Dihuang pill had the highest comprehensive ranking for the use and frequency of CPM from 2008 to 2010 [29]. Regarding CHM decoction, a cohort study involving 154,083 people in Taiwan from 2003 to 2009 showed that about 80% of patients used traditional Chinese medicine at least once. Tianma Gouteng decoction and salvia miltiorrhiza were the most frequently used Chinese medicine [18]. From 1996 to 2005, the main herbal medicine types for hypertension in the Beijing area were tonify deficiency medicine, levelling liver and calming wind drugs, heat-clearing drugs, blood-activating and stasis-eliminating compound, and damp-clearing drugs [30]. Similarly, the study found that in the past 30 years, the first 5 effective treatments were activating the blood and dissolving stasis, xifeng antispasmodic, benefit qi and blood, smooth liver yang, and removal of pathogenic heat from the blood [31]. In summary, there was a consistent use of medication for hypertension prescriptions.

### 3.8. TCM for the Treatment of Hypertension and Complications

**Figure 7:**
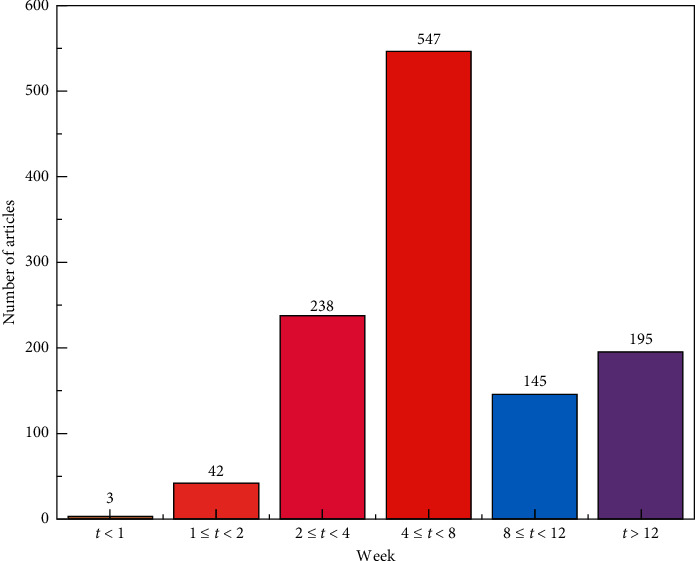
Duration distribution of TCM intervention in hypertension.

The current main research target is the middle-aged and elderly hypertension and mainly involves grade 1–2 hypertension. A total of 1,214 studies focussed on elderly hypertension and 42 studies focussed on the treatment of middle-aged and young patients with hypertension. A total of 2,579 studies focussed on hypertension and its complications, accounting for 27.43% of the total research. The top 3 complications were intracerebral haemorrhage (*n* = 693, 26.9%), kidney damage (*n* = 397, 15.4%), and diabetes mellitus/abnormal glucose (*n* = 378, 14.7%) (Table 2). A total of 1,309 articles commonly used intervention of traditional Chinese medicine preparations and 1,170 articles used the analysis intervention duration, most of which were concentrated in a 4–8 week period (*n* = 547, 46.8%), of which only 3 articles of more than 42 months of intervention were present in the strongly exposed group [32–34], suggesting that the research time limit of TCM intervention in hypertension was generally shorter (Figure 7).

### 3.9. Evidence Quality and Evaluation of the Included Systematic Reviews

**Figure 8:**
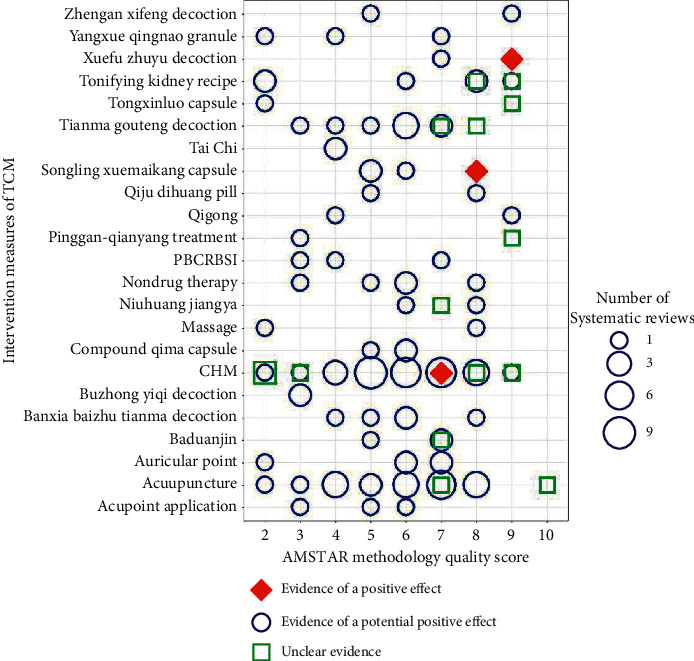
Evidence distribution diagram of systematic reviews. The plot depicts the estimated number of SRs (size of the bubble), the clinical efficacy of SRs (shape and colour of the bubble), the AMSTAR scores (*x* axis), and the types of TCM intervention (*y* axis). Green squares indicate unclear evidence of SRs, blue bubbles indicate potential evidence of SRs, and red rhombus indicate positive evidence of SRs. PBCRBSI: promoting blood circulation and removing blood stasis injection.

A total of 144 systematic reviews were retrieved; there were 5 overviews of SRs without analysis [35–39]. The evidence map for TCM is based on the 139 published systematic reviews, including the Chinese herbal medicine studies (*n* = 92) and nondrug therapy studies (*n* = 47). The single CHM and nondrug therapy were combined into one category, respectively. According to the types of TCM intervention, the intervention principles were divided into 23 types: acupuncture (*n* = 24), qigong (*n* = 2), Tai Chi (*n* = 2), baduanjin (*n* = 4), massage (*n* = 2), auricular point (*n* = 5), acupoint application (*n* = 3), songling xuemai kang capsule (*n* = 4), tongxinluo capsule (*n* = 2), yangxueqingnao granule (*n* = 3), tianmagouteng decoction (*n* = 11), niuhuang jiangya (*n* = 4), banxia baizhu tianma decoction (*n* = 5), buzhong yiqi decoction (*n* = 2), xuefu zhuyu decoction (*n* = 2), Promoting blood circulation and removing blood stasis injection (PBCRBSI) (*n* = 3), pinggan-qianyang treatment (*n* = 2), qiju dihuang pill (*n* = 2), compound qi ma capsule (*n* = 3), tongxinluo capsule (*n* = 2), zhengan xifeng decoction (*n* = 2), tonifying kidney herbs (*n* = 8), CHM (*n* = 40), and nondrug therapy of TCM (*n* = 4). The quality of the included reviews is shown in Figure 8.

According to the AMSTAR scale evaluation, the most qualified item, 9, had 138 SRs. However, 137 SRs did not provide the preliminary design scheme, 106 reviews did not consider the retrieval and inclusion of the grey literature, 70 SRs did not perform a comprehensive literature search, 29 SRs did not properly apply the scientific quality of the included studies to the derivation of conclusions, 31 SRs did not assess and document the scientific quality of the included studies, 138 SRs did not provide the list of included and excluded research literature, 40 SRs did not assess the likelihood of publication bias assessment, and 117 SRs did not provide a conflict of interest statement. One review met 10 criteria [40] and nine reviews met 9 criteria [6, 7, 41–47]. The authors considered these 10 systematic reviews to be of high quality. A total of 94 systematic reviews were of moderate quality and met the 8 AMSTAR criteria (*n* = 19), 7 criteria (*n* = 27), 6 criteria (*n* = 27), and 5 criteria (*n* = 21). The other 35 systematic reviews were of the lower quality and met 4 criteria (*n* = 14), 3 criteria (*n* = 10), or 2 criteria (*n* = 11).

Regarding clinical evidence with SRs, a small number of SRs had unclear evidence (*n* = 16) [40, 42, 43, 45, 47–58]. Most of the SRs had a potentially positive effect (*n* = 120) [7, 44, 46, 59–114], concentrated in 5–8 score. Three SRs were positive, concentrated in the 7–9 score. [6, 115, 116]. To summarise, most of the included SRs were based on the poor quality of primary studies and the quality of clinical efficacy of most primary outcomes was a potentially positive effect (86%).

### 3.10. A General Overview of the Systematic Reviews

#### 3.10.1. CHM plus Antihypertensive Drugs versus Antihypertensive Drugs

In the 139 SRs, most of the intervention measures were CHM combined with Western medicine (77, 55.4%). Forty-three SRs (quality range = 2–8) included SBP as an outcome measure, 38 SRs (quality range = 2–9) included DBP as an outcome measure, 20 SRs (quality range = 2–8) included total effective rate as an outcome measure, and 18 SRs (quality range = 4–9) included TCM-SSD scores as an outcome measure. There are significant differences in the effect of CHM plus antihypertensive drugs for lowering SBP (*n* = 35, 81.4%) [6, 41, 43, 45, 46, 58, 61, 62, 64, 65, 67–69, 78, 80, 92, 93, 95, 96, 99, 101, 102, 114, 117–127], lowering DBP (*n* = 26, 68.4%) [6, 41, 45, 46, 58, 61, 62, 64, 68, 69, 78, 80, 93, 95, 101, 102, 119, 121–126], improving total effective rate (*n* = 18, 90.0%) [58, 76, 82, 83, 99,103, 107, 108, 111, 112, 114, 121, 122, 126, 128–131], and lowering TCM-SSD scores (*n* = 17, 94.4%) [7, 41, 46, 48, 62, 76, 79, 94, 95, 99, 102, 112, 130, 132–135] than the antihypertensive drugs. The Xinmaitong (6 RCTs; quality = 7) and songling xuemakang capsules (4 RCTs; quality = 8), combined with antihypertensive drugs, significantly lowered the SDP and DBP and improved clinical efficacy, with low heterogeneity. Clinical evidence was the recommended level [115, 116].

#### 3.10.2. CHM versus Antihypertensive Drugs

Among the 139 SRs, 42 SRs (27.3%) were of the CHM therapy alone. The outcome measures SBP, DBP, total effective rate, and TCM-SSD scores included 21 SRs, 20 SRs, 13 SRs, and 10 SRs, respectively. There were significant differences in the effect of CHM for lowering SBP (*n* = 12, 57.1%) [7, 45, 58, 62, 92, 93, 102, 116, 121, 127, 136, 137], lowering DBP (*n* = 7, 35.0%) [7, 45, 62, 93, 102, 116, 137], improving the total effective rate (*n* = 6, 46.1%) [55, 81, 110, 130, 138, 139], and lowering the TCM-SSD scores (*n* = 9, 90.0%) [7, 48, 62, 84, 94, 102, 112, 115, 133].

#### 3.10.3. Nondrug Therapy plus Antihypertensive Drugs versus Antihypertensive Drugs

Of the 139 SRs, 38 SRs (27.3%) were nondrug therapy combined with Western medicine. The outcome measures of SBP, DBP, total effective rate, and TCM-SSD scores were evaluated separately in 31 SRs, 26 SRs, 17 SRs, and 6 SRs. There were significant differences in the effect of nondrug paratherapy for lowering the SBP (*n* = 28, 90.3%) [44, 53, 59, 63, 66, 71, 73, 74, 87, 91, 105, 106, 113, 140–153], lowering DBP (*n* = 22, 84.6%) [44, 53, 59, 63, 66, 71, 73, 74, 105, 140–151], improving the total effective rate (*n* = 16, 94.1%) [71, 90, 91, 113, 140, 142, 143, 145, 147, 148, 150, 152, 154–157], and lowering the TCM-SSD scores (*n* = 6, 100.0%) [70, 71, 105, 152, 156].

#### 3.10.4. Nondrug Therapy versus Antihypertensive Drugs

Of the 139 SRs, 24 SRs (17.3%) involved nondrug therapy. The outcome measures SBP, DBP, total effective rate, and TCM-SSD scores were evaluated in 14 SRs, 12 SRs, 8 SRs, and 6 SRs, respectively. There were significant differences in the effect of nondrug therapy for lowering the SBP (*n* = 6, 42.8%) [44, 53, 91, 105, 146, 151], lowering DBP (*n* = 6, 50.0%) [44, 53, 105, 113, 141, 146], improving the total effective rate (*n* = 4, 50.0%) [91, 158–160], and lowering the TCM-SSD scores (*n* = 5, 83.3%) [70, 88, 105, 156, 160].

### 3.11. Potentially Promising Effects in High-Quality (AMSTAR ≥9) Literature

A total of 10 high-quality studies were retrieved, where qigong (20 RCTs), zhengan xifeng decoction (6 RCTs), Liuwei Dihuang pill (6 RCTs), and CHM (24 RCTs) were considered to have evidence of potential positive effects. Xuefu zhuyu decoction (15 RCTs) was considered a positive effect; i.e., we are confident in estimating the research results. The SRs of acupuncture (22 RCTs), shenqi pill (4 RCTs), jianling decoction (10 RCTs), tongxinluo capsule (25 RCTs), and CHM (5 RCTs) had unclear evidence.

### 3.12. Blood Pressure

#### 3.12.1. CHM versus Antihypertensive Drugs

Zhengan xifeng decoction showed a significant difference in the SBP and DBP control (*P* < 0.05; 4 RCTs) compared to antihypertensive drugs [7]. In contrast, one SR showed no significant differences [43].

#### 3.12.2. CHM plus Antihypertensive Drugs versus Antihypertensive Drugs

The pooled results of the largest review (24 RCTs, 4502 participants) showed a high number of participants with reduced blood pressure (relative risk (RR) 1.28; 95% confidence interval (CI) 1.21, 1.36, *P* < 0.001; 8 RCTs (RR: 1.12; 95% CI 1.06, 1.39, *P* < 0.001; 5 RCTs)). However, the authors cautioned evidence of a potentially positive effect due to the poor quality of the included RCTs [41]. Fifteen studies reported significant effects of xuefu zhuyu decoction combined with antihypertensive drugs (15 RCTs, 1364 participants) for lowering the blood pressure compared to the control group (*P* < 0.05). The author suggested that xuefu zhuyu decoction for hypertension should be prioritised for future preclinical and clinical studies [6]. The Liuwei Dihuag pill (6 RCTs, 555 participants) and jian ling decoction combined with antihypertensive drugs were more effective in controlling the blood pressure [43, 46]. In contrast, 2 SRs showed no significant difference [42, 47].

#### 3.12.3. Nondrug Therapy plus Antihypertensive Drugs versus Antihypertensive Drugs

Qigong plus antihypertensive drugs significantly lowered both the SBP (WMD = -11.99 mmHg; 95% CI −15.59, −8.39, *P* < 0.00001) and DBP (WMD = -5.28 mmHg; 95% CI −8.13, −2.42, *P*=0.0003; 5 RCTs) compared to the antihypertensive drugs alone. Compared to no intervention, qigong significantly reduced SBP and DBP (*P* < 0.05) [44]. One Cochrane review concluded that the clinical evidence for short-term and sustained BP-lowering effect by acupuncture was unclear (quality = 10) [40].

### 3.13. TCM-SSD Scores

#### 3.13.1. CHM versus Antihypertensive Drugs

One SR showed a significant effect of CHM for lowering the TCM-SSD scores compared to the antihypertensive drugs [7].

#### 3.13.2. CHM plus Antihypertensive Drugs versus Antihypertensive Drugs

Two SRs showed a significant effect of CHM combined with antihypertensive drugs for lowering the TCM-SSD scores compared to the antihypertensive drugs [41, 46].

### 3.14. Adverse Events

Of the 139 SRs, there was an outcome measure of adverse effects in 77 SRs, which included gastrointestinal reaction, dizziness, headache, cough, and nausea [41, 49]. In summary, all of these SRs indicated that the side effects in the TCM adjuvant therapy group were generally less than or lighter than those in the Western medicine group.

#### 3.14.1. Guidelines, Consensus, and Clinical Pathway Studies

A total of 16 papers were retrieved on the treatment of hypertension guidelines, consensus, and clinical pathway of TCM research, including the TCM treatment for hypertension and its complications and consensus (*n* = 10), consensus recommendation on the application for CPM of hypertension (*n* = 1) [161], the optimisation path of TCM clinical program (*n* = 4), and the nursing clinical path (*n* = 1) [162]. In 2019, more than 70% of the experts recommended 6 types of CPM: the Tianma Gouteng decoction, qiju dihuang capsule, jinguishenqi pill, gingko leaf tablets, niuhuang jiangya pill, and banxia tianma pill to help non-TCM practitioners to select appropriate CPM according to the TCM symptoms. In addition, multicentre RWS found that the 7 common syndromes under the TCM diagnosis and treatment guidelines for hypertension, including liver fire flaming upward syndrome, yin deficiency and yang hyperactive syndrome, blood stasis and internal obstruction, phlegm and dampness, deficiency of qi and blood, deficiency of kidney essence, and chong and ren imbalance, only accounted for 58.38% of the common syndromes. Further, adding the phlegm and blood stasis mutual settlement syndrome is recommended and so it cancels the chong and ren imbalance [163]. Guidelines and path research guide the treatment of EH in TCM and also guide the treatment of complications such as acute cerebral haemorrhage and depression.

## 4. Discussion

In this study, an evidence map was used to systematically sort the literature on hypertension in the past 20 years. Compared to the previous evidence mapping studies that only included RCTs or SRs [11–14], the current study mainly focussed on the diversified research types (observational studies, interventional studies, secondary studies, and RWS), intervention measures (CHM and nondrug therapy), and the analysis contents (TCM prevention and treatment schemes, intervention time, study outcomes, adverse reactions, etc.) has been expanded to provide a comprehensive description of the clinical problem. It shows the volume and field of available research and highlights areas where published meta-analysis has reported positive results and identified gaps in evidence.

### 4.1. Advantages of TCM in the Prevention and Treatment of Hypertension

For hypertension prevention and treatment by TCM, the key areas to target are lowering BP, lowering the TCM-SSD scores, improving the clinical symptoms, and protecting the target organs. The adverse events in the TCM paratherapy group were generally less than those in the control group. A total of 120 SRs found that CHM and nondrug therapy had potential active effects for the treatment of hypertension, 16 SRs showed unclear evidence, and 3 SRs showed active effects. Regarding complications, damage to the heart, brain, and kidney target organs accounted for more than 50% of the studies, and TCM had a good effect on the dissipation of the hypertensive cerebral haematoma, stroke score, proteinuria, and left ventricular hypertrophy. Meanwhile, the evaluation of TCM clinical programs showed that TCM combined with Western medicine can enhance clinical effectiveness and reduce adverse events. Regarding clinical symptoms, it had an improved effect on the main symptoms of vertigo, headache, and systemic symptoms. Based on the study of guidelines and pathways, TCM syndromes and CPM (tianma gouteng decoction qiju dihuang capsule, jingui shenqi pill, gingko leaf tablets, niuhuang jiangya pill, and banxia tianma pill) have been put forward for clinical application.

### 4.2. Future Focus on TCM Prevention and Treatment of Hypertension

TCM intervention for prehypertension is still insufficient. At present, only 3 SRs have been published, including nondrug therapy (17 RCTs, quality = 6) [160], CHM (8 RCTs, quality = 5) [137], and CHM (5 RCTs, quality = 8) [51]. In the future, greater focus should be placed on improving prevention and treatment during early hypertension, including prehypertension, grade 1 hypertension, and youth hypertension, and additional research should be carried out on specific clinical indicators and mechanisms. It is also important to investigate in emotion, obesity, and other hypertension risk factors by CHM and nondrug therapy.

### 4.3. Limitations and Implications

In general, a summary of the findings of included SRs and clinical studies showed that TCM paratherapy for EH has better efficacy and safety than the control group. The research evidence on the risk factors, quality of life, emotional and psychological, early intervention, duration of intervention, and adverse events is weak. However, there are several limitations to the present study. First, the evidence map provides only a broad overview of the research areas and cannot provide definitive answers regarding the effectiveness of an intervention. The specific control of clinical indicators requires more detailed and targeted research. Second, the evidence map did not establish the reporting guidelines and did not avoid overlap between the included studies across reviews. Third, the quality of the methodology of most SRs was low (25.2%) to moderate (67.6%), which directly influences the reliability of the results. Fourth, literature types, heterogeneity, and complex intervention measures in the included studies only elucidate the efficacy and safety at a macroscopic level.

The improvements for further evidence map are as follows [164, 165]. In terms of data sources, a complementary search of the clinical registration platform and references should be additionally conducted. Regarding content extraction, one should further focus on the retrieval according to the priority areas to further improve accuracy. To avoid unrecognised individual literature due to a large number of retrieved literature and the problem of splitting the same research results, topic selection of TCM literature should focus on specific clinical problems, avoid extensive titles, and prevent the result from being too complex for an explanation. Finally, one should review the evidence base with standard evidence synthesis methods (i.e., systematic review), improve the methodological quality of SRs themselves, and encourage prospective registration of SRs.

## 5. Conclusion

The conclusion of the SRs and primary studies highlight TCM's advantages as adjunctive therapy for improving hypertension. Similarly, the development trend of CHM and nondrug therapy for the prevention and treatment of hypertension is relatively good, which reflects the diverse TCM prevention and treatment measures for hypertension. However, clinical research evidence needs to be treated with caution because of methodological flaws. In the future, studies with larger sample sizes, standardisation, and higher quality are required to provide further scientific evidence for TCM in treating hypertension.

## Figures and Tables

**Table 1:** Clinical study scale.

Study sample size (*n*)	Number of research articles (*n* (%))		
	Intervention study	Observational study	Real-world study
*n*＜60	1252 (15.54）	41 (3.46)	0
60≤*n*＜100	3977 (49.38）	146 (12.32)	0
100≤*n*＜300	2638 (32.76)	595 (50.21)	0
300≤*n*＜1000	167 (2.07)	316 (26.67)	0
*n*≥1000	19 (0.24)	87 (7.34)	5 (100.00)
Total	8053	1185	5

**Table 2:** Distribution of research on prevention and treatment of hypertension and the complications by TCM.

Complication	Number of research articles (*n* (%))	
Cerebral haemorrhage	693	26.9
Kidney damage	397	15.4
Diabetes mellitus/abnormal glucose metabolism	378	14.7
Left ventricular dysfunction	173	6.7
Hyperlipidaemia	132	5.1
Sleep disorder	125	4.9
Angina	113	4.4
Atherosclerosis	107	4.2
Anxiety and depression	105	4.1
Cerebral infarction	76	2.9
Disease of the eyes	45	1.8
Arrhythmia	40	1.6
Hyperuricemia	27	1.1
Metabolic syndrome	22	0.9
Ventricular dysfunction	7	0.3
Hyperviscositemia	3	0.1
Others	136	5.3

## Data Availability

The datasets used during the current study are available from the corresponding author upon reasonable request.
